# Machine learning-based identification of genetic interactions from heterogeneous gene expression profiles

**DOI:** 10.1371/journal.pone.0201056

**Published:** 2018-07-26

**Authors:** Chihyun Park, JungRim Kim, Jeongwoo Kim, Sanghyun Park

**Affiliations:** Dept. of Computer Science, Yonsei University, Seodaemun-gu, Seoul, Korea; Indraprastha Institute of Information Technology, INDIA

## Abstract

The identification of disease-related genes and disease mechanisms is an important research goal; many studies have approached this problem by analysing genetic networks based on gene expression profiles and interaction datasets. To construct a gene network, correlations or associations among pairs of genes must be obtained. However, when gene expression data are heterogeneous with high levels of noise for samples assigned to the same condition, it is difficult to accurately determine whether a gene pair represents a significant gene–gene interaction (GGI). In order to solve this problem, we proposed a random forest-based method to classify significant GGIs from gene expression data. To train the model, we defined novel feature sets and utilised various high-confidence interactome datasets to deduce the correct answer set from known disease-specific genes. Using Alzheimer’s disease data, the proposed method showed remarkable accuracy, and the GGIs established in the analysis can be used to build a meaningful genetic network that can explain the mechanisms underlying Alzheimer’s disease.

## Introduction

For a comprehensive understanding of complex disease mechanisms, network approaches are widely [1–3]. These biological networks can contain physical or genetic interactions. A representative physical network is protein–protein interactions. Although there are various types of genetic interaction networks with different properties, their basic role is to model relationships among molecules in order to identify and explain underlying biological processes or functional dynamics related to a disease or phenotype [4].

The most important step in the construction of a genetic interaction network is the extraction of gene–gene interactions (GGIs) from omics data profiles. Many approaches have been proposed to identify GGIs [5–7]. In particular, incorporating interactome and transcriptome data has proven to be useful for the extraction of co-expressed GGIs [8]. A novel approach for calculating the strength of interactions with significantly different correlations has been proposed [9]. Using this approach, cancer-specific gene network has been derived and it applied to classify cancer.

The final goal of many approaches for GGI identification is to construct disease-specific gene networks and apply them to reveal disease-related targets and mechanisms. In cancer research, this kind of network biology approach is widely used [3, 10–11]. In Alzheimer’s disease (AD) research, several integrative approaches using gene expression and interactome datasets have been proposed to infer genetic networks [12–15].

Based on previous research, the most popular method to identify significant GGIs is to measure the correlation coefficient from two gene expression vectors. Because there are many similarity measures, it is important to determine the most appropriate measure for a particular dataset. In a recent study, 12 frequently used correlation measures were compared to identify the optimal approach for extracting functional information from gene expression profiles [16]. The authors concluded that linear similarity measures, such as the dot product or Pearson's correlation coefficient (PCC), or cosine similarity performed better than other similarity measures, including set overlap measures, such as the Jaccard coefficient. The authors also demonstrated that the dot product showed the most consistent performance for the gene expression dataset, which had noise and batch-effects [16]. However, it is difficult to accurately measure correlations by linear similarity approaches when data include high levels of noise and heterogeneity.

A recent study attempted to use non-linear correlation measures, such as mutual information (MI), to extract differential co-expressed GGIs from heterogeneous gene expression data [17]. This study aimed to construct AD-specific genetic networks, despite heterogeneity in expression levels across large samples. AD is known as a clinically heterogeneous neurodegenerative disease; furthermore, the underlying genetic factors and their functional roles have not been revealed [18]. As a result of the heterogeneity, the sample quality among patients with AD and the affected degree of gene expression may be inconsistent. Recently, a study has attempted to identify heterogeneous genes from AD gene expression data [19].

**Fig 1:**
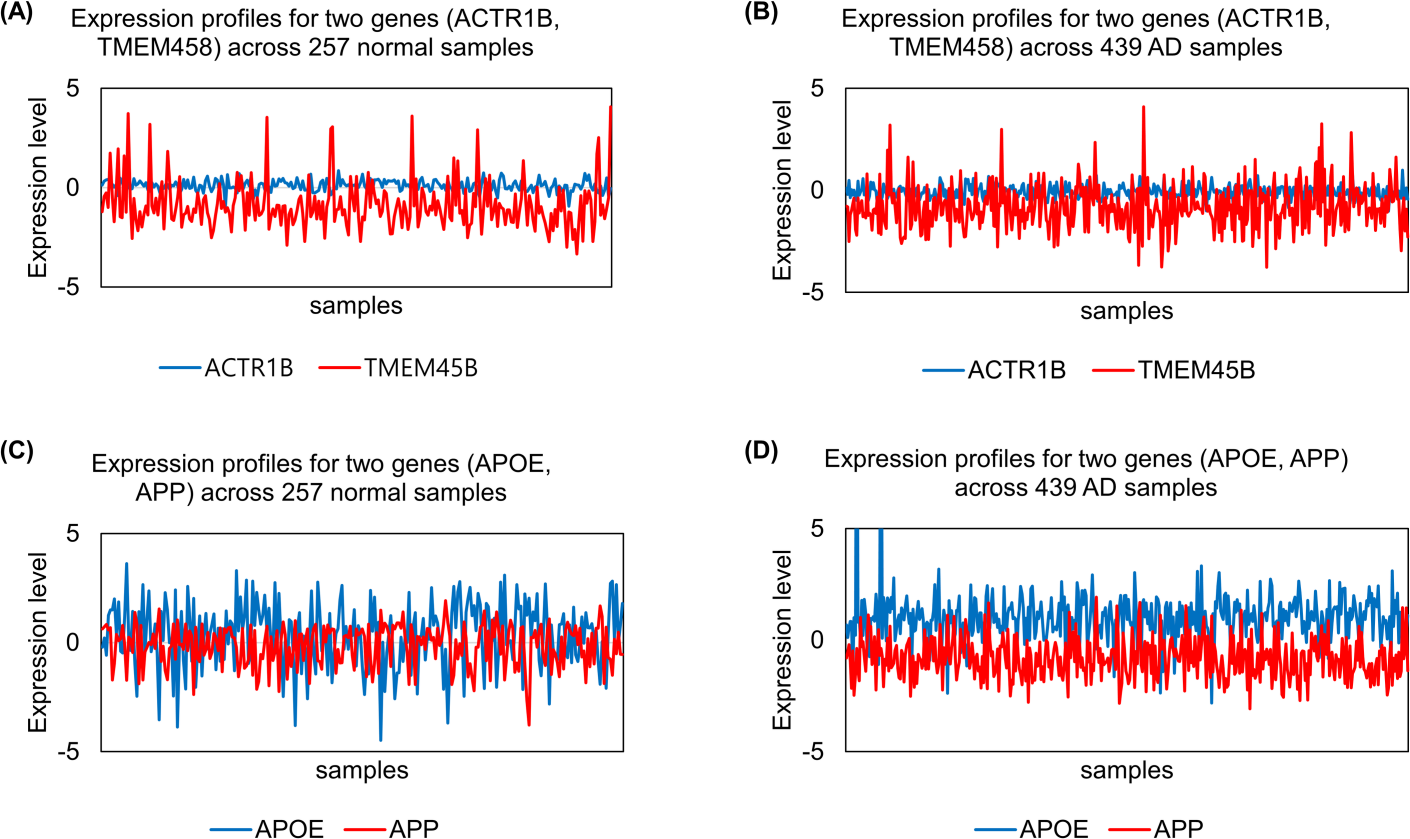
Visualisation of expression levels for four genes according to their class label (Normal and AD). Four genes were divided into two groups, i.e. AD-unrelated and -related groups.

In a study using a non-linear similarity measure [17], it was challenging to extract informative GGIs. For a specific explanation, we randomly selected expression data for *ACTR1B*, *TMEM45B*, *APOE*, and *APP* from real datasets (GSE33000, GSE44770) and applied the z-scoring method for normalisation. Then, we visualised these expression values and calculated mean, standard deviation, and PCC values, as shown in (Fig 1) and Table 1. These four genes can be divided into two groups based on previous studies, i.e. AD-related and AD-unrelated. As shown in (Fig 1) and Table 1, because the expression values were heterogeneous across samples, it was not adequate to determine whether known AD-related genes had a stronger association with AD than normal samples and whether AD-unrelated genes have a stronger association in normal than AD samples based on the PCC value. In this case, it is consequentially difficult to determine the appropriate threshold for extracting meaningful GGIs to build a genetic network.

**Table 1:** Basic statistics and PCC values for four cases shown in Fig 1. The correlation values for AD-related genes were relatively larger than those for AD-unrelated genes. However, the correlation values for AD-related genes were not sufficient to accurately determine correlations in AD.

AD-unrelated genes	Case (see Fig 1)	Class label	Gene	Mean of expression values	Standard deviation of expression values	PCC of two expression lists
	(A)	Normal	*ACTR1B*	0.099	0.284	0.021
			*TMEM45B*	-0.882	1.207	
	(B)	AD	*ACTR1B*	-0.070	0.304	-0.080
			*TMEM45B*	-0.832	1.094	
AD-related genes	(C)	Normal	*APOE*	0.359	1.475	-0.590
			*APP*	-0.135	0.952	
	(D)	AD	*APOE*	0.997	1.369	-0.280
			*APP*	-0.817	0.906	

As in the example above, using correlations or similarity measures exclusively may not be appropriate to extract GGIs, depending on the properties of the dataset, such as heterogeneity across samples. In this case, machine learning-based approaches can be an alternative [7]. According to a recent review study [7], typical machine learning approaches, such as artificial neural network (ANN), support vector machine (SVM), and random forest, have been widely applied to detect GGIs. This paper established that a random forest-based approach is suitable for datasets with genetic heterogeneity.

In this study, we propose a novel approach to build a machine learning-based model that can determine significant GGIs from heterogeneous gene expression profiles. We designed a novel feature set from expression profiles and utilised various interactome datasets and gene sets known to be associated with a disease in order to assign a label for gene pairs. We demonstrated that our approach shows remarkable performance in the case of AD with large-scale expression data.

## Materials and methods

**Fig 2:**
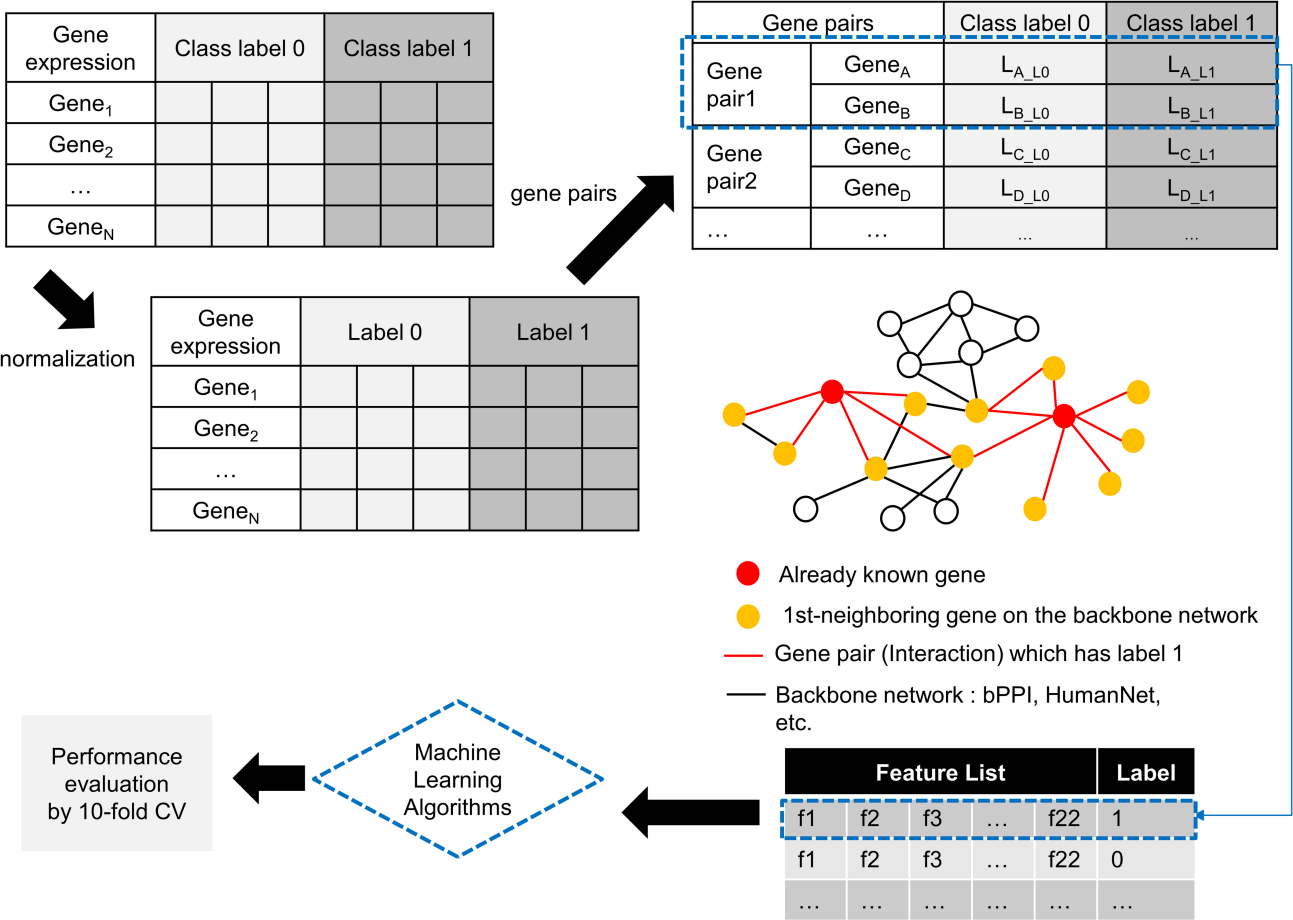
Overview of the proposed approach. Gene expression data with two class labels are normalized by the z-scoring approach. For class label 1, which indicates disease, possible gene pairs are selected by incorporating disease-related genes and interactome data. For class label 0, which indicates normal, the same number of gene pairs as that for class label 1 is randomly selected. From all gene pairs, 22 features are extracted and used to inform the machine learning-based model. In order to evaluate performance, 10-fold cross validation is performed.

In this section, we introduce the entire approach with an explanation of how we formulated GGI identification as a machine learning problem, and then present the detailed procedures. As shown in (Fig 2), normalization and data transformation by feature extraction were performed and a machine learning algorithm was applied.

### Datasets

We used two recently published large-scale gene expression profiles [20–21]. The datasets were obtained by human brain tissue sampling to investigate the mechanism underlying late-onset AD. Focusing exclusively on the prefrontal cortex, we integrated these two expression profiles (GSE33000 and GSE44770) to increase the sample size; this was possible because the same platform was used to generate both datasets. The integrated dataset was composed of 257 non-demented, i.e. normal, and 439 AD samples.

An interactome dataset and disease-related gene set were used to label the gene pairs. We utilized two AD-related data sources. The first was the AD-associated gene network curated by the IntAct database [22] and the second was AD-related genes identified in a genome-wide association study (GWAS) [23].

Along with these two datasets, we used two interactome datasets, a human protein interaction dataset [24] and HumanNet [25]. The first dataset was composed of 23,233 high-confidence interactions identified by systematic screening based on high-throughput yeast two-hybrid experiments and validated using biological assays. These data are referred to as biophysical protein–protein interactions (bPPI). The second dataset was constructed by the large-scale integration of co-expressed and/or co-occurring gene pairs using many sources. To obtain more accurate and biologically meaningful interactions, we used bPPI alone or integrated bPPI with the top 5 or 10 percent of interactions in accordance with confidence scores of interactions in humanNet.

### GGI identification with machine learning

Instead of measuring the correlation values for all possible gene pairs from the gene expression profile, we assume that it may be more effective to obtain GGIs by learning the expression patterns of gene pairs known to be specific to AD. In other words, the learning model can classify whether a gene pair is informative or not based on its expression pattern by referring to the expression pattern of gene pairs already known to be AD-specific. As mentioned above, if the expression profile is highly heterogeneous, there is a high probability of that the correlation values for gene pairs is not sufficient. Other gene pairs tend to follow the expression pattern for potent GGIs already known to be associated with AD. Therefore, if we have enough expression datasets to make a model, and if there is a gene or gene network already known for a certain disease, we can formulate it to a machine learning problem.

#### Definition of features

To define features from an expression profile, we use various statistical measurements. Because we assume that gene expression data have disease and normal statuses, each gene pair can be represented as shown in Table 2.

**Table 2:** Notation of gene expression values for each class and gene in one gene pair.

Gene pair	Class label 0 (Normal)	Class label 1 (AD)
Gene A	E_A_L0_	E_A_L1_
Gene B	E_B_L0_	E_B_L1_

E_A_L0_ denotes the expression value list for gene A of samples labelled 0. Similarly, E_B_L1_ indicates the expression value list for gene B of samples labelled 1. We extract 22 features from these four expression value lists. Table 3 shows the list of features. Basic statistics, such as the mean or standard deviation, are included first. Then, the differences between maximum elements and minimum elements are calculated for each expression value list, E. Despite the use of means and standard deviation, the difference value is added to better reflect the heterogeneity. In addition, the statistics for Welch’s *t*-test are included in the feature list to reflect the difference between two groups. According to Ruxton [26], Welch’s *t*-test is more reliable when two samples have unequal variances and unequal sample sizes. This property is particularly suitable for the comparison between E_A_L0_ and E_A_L1_ or E_B_L0_ and E_B_L1_.

**Table 3:** List of the features.

Feature name	Definition
Mean_A_L0_	mean of E_A_L0_
Mean_A_L1_	mean of E_A_L1_
Mean_B_L0_	mean of E_B_L0_
Mean_B_L1_	mean of E_B_L1_
SD_A_L0_	standard deviation of E_A_L0_
SD_A_L1_	standard deviation of E_A_L1_
SD_B_L0_	standard deviation of E_B_L0_
SD_B_L1_	standard deviation of E_B_L1_
dMm_A_L0_	maximum element of E_A_L0_ –minimum element of E_A_L0_
dMm_A_L1_	maximum element of E_A_L1_ –minimum element of E_A_L1_
dMm_B_L0_	maximum element of E_B_L0_ –minimum element of E_B_L0_
dMm_B_L1_	maximum element of E_B_L1_ –minimum element of E_B_L1_
WT_A_L0_B_L0_	Welch’s *t*-test statistics (E_A_L0_, E_B_L0_)
WT_A_L1_B_L1_	Welch’s *t*-test statistics (E_A_L1_, E_B_L1_)
WT_A_L0_A_L1_	Welch’s *t*-test statistics (E_A_L0_, E_A_L1_)
WT_B_L0_B_L1_	Welch’s *t*-test statistics (E_B_L0_, E_B_L1_)
PCC_A_L0_B_L0_	Pearson's correlation coefficient (E_A_L0_, E_B_L0_)
PCC_A_L1_B_L1_	Pearson's correlation coefficient (E_A_L1_, E_B_L1_)
MI_A_L0_B_L0_	Mutual Information (E_A_L0_, E_B_L0_)
MI_A_L1_B_L1_	Mutual Information (E_A_L1_, E_B_L1_)
MI_A_L0_A_L1_	Mutual Information of Make equal-sized element list (E’_A_L0_, E’_A_L1_)
MI_B_L0_B_L1_	Mutual Information of Make equal-sized element list (E’_B_L0_, E’_B_L1_)

We also apply two correlation-based similarity measures, PCC and MI. The correlation between two element lists corresponding to two genes and labelled as belonging to the same class is computed. Moreover, the correlation between two element lists labelled as belonging to different classes, but corresponding to the same gene is computed. In this case, owing to an imbalance in the elements size of two lists, an under-sampling approach is used. We denote the under-sampling element list as E′. For example, let E_A_L0_ and E_A_L1_ be [1,2,3,4,5] and [6,7,8], respectively. To calculate MI for these two element lists, undersampling is performed by randomly pulling elements so that the size of E_A_L0_ is equal to that of E_A_L1_. For example, after sampling, E_A_L0_ can be [1,4,5]. The detail of calculating Welch’s *t*-test statistics and MI are described in the supplementary material.

#### Assigning labels to gene pairs

In order to build a supervised learning model, labels should be assigned to the training dataset. Labels are assigned using the interactome and known disease genes. The most important data for the correct answer set is the AD-related gene network identified by the IntAct database. However, these data are only composed of approximately 360 GGIs, which it is not enough to train the model. Therefore, we suggest a method to boost the correct answer set. In particular, we applied a method that can extract the k-nearest neighbour gene from the interactome using a gene known to be associated with AD as a seed. Because indirect effects likely applies to neighbouring genes from the seed in the interactome network, this training data set can be further extended to include these GGIs.

#### Random forest

The optimal machine learning algorithm is not clear because the type of dataset and issues associated with GGI detection vary [7]. In this study, since the genetic and sample heterogeneity are the main problems, we selected a random forest algorithm. Random forest is particularly useful for addressing genetic heterogeneity because subsets of the model are separated in the early stage [7]. For example, if an input data consist of *N* instances with *M* features, then random forest algorithm randomly selects some of *N* and *M* and builds decision tree. The random forest algorithm iteratively performs this task to build many decision trees. In this process, each independent model, i.e. decision tree, is learned to fit for subset of input data. As a result, arbitrarily selected features may not affect predictive performance if they are heterogeneous or may significantly affect predictive performance if they are not. Through randomization in learning stage, the features with strongly predictive performance are continuously selected to improve overall performance. In addition, random forest avoids overfitting the data. The random forest algorithm used in the study is built by the pseudocode summarised in Algorithm 1. After building the classifier, the unlabelled instance is introduced to the randomly created trees from the random forest. Then, the classified results are aggregated and the highest index value is used to determine the final result.

**Algorithm 1.** Build Random Forest (Input, Parameters)

Description

Build random forest algorithms with input data

Input

Training data *D* consists of sample *S*, features *F*, and label *y*

Parameters

Preferred number of instances *n* = 100

Number of trees *t* = 100

Output

Trained classification model

1. Randomly select *n* instances with *k* (= log_2_*t*) features from *F*. Set this to *P*. (*P*⊆*D*)
2. With *P*, among the *k* features, calculate node *d* using the best split point
3. Split the node into daughter nodes using the best split
4. Repeat 1 to 3 steps until the three is formed with a root and a target as the leaf node
5. Build forest by repeating steps 1 to 4 for *t* times to create *t* trees.

## Results and discussion

### Evaluation and performance comparison

We performed various tests to compare the proposed algorithm with typical machine learning algorithms, while changing the dataset. As mentioned above, in case of AD, it is difficult to accurately determine whether there is an interaction between two genes because expression values for one gene can be heterogeneous even in samples with the same label. This was the reason why we determined to focus on AD as a targeted disease. Throughout the evaluation, we tried to determine which interactome data should be used to build an effective classification model and whether AD-related genes could be used as a seed to improve the performance of the learning model. To achieve these aims, we prepared various datasets for comparative analyses. A detailed description of the datasets and the comparative algorithms are provided in Tables 4 and 5, respectively.

**Table 4:** Detailed description of the dataset used for performance evaluation. For all datasets, we used the AD-gene network published by the IntAct Molecular Interaction Database, which is curated by broad literature searches. However, since the size of the IntAct(AD) was small, interactome data were integrated to increase the size of the training dataset.

Dataset ID	Description of dataset		Sample Size (number of interactions)	
	Interactome dataset	Use of AD-related genes (seed gene)	Normal (class label 0)	AD (class label 1)
1	IntAct(AD) + bPPI	Y	3,241	3,241
2	IntAct(AD) + bPPI + HumanNet (5%)	Y	4,916	4,916
3	IntAct(AD) + bPPI + HumanNet (10%)	Y	7,013	7,013
4	IntAct(AD) + bPPI	N	23,546	23,546
5	IntAct(AD) + bPPI + HumanNet (5%)	N	46,206	46,206
6	IntAct(AD) + bPPI + HumanNet (10%)	N	69,296	69,296

**Table 5:** List of the comparative algorithms and their primary parameters.

Algorithms	Mainly used options
Naïve Bayes [27]	No parameters
SVM [28]	polynomial kernel complexity = 1.0 epsilon = 1.0E-12 tolerance = 0.001
ANN [29] (Multi-Layer Perceptron)	hidden layer = 3 learning rate = 0.3 momentum = 0.2 number of epochs = 200
PART [30]	minimum number of instances per rule = 2 confidence factor used for pruning = 0.25, seed = 1

Let us assume that if there are 20,000 genes, the entire number of possible gene pairs is greater than about 199 million. If we use all possible gene pairs for learning, a severely imbalanced distribution of labels may occur because the gene pairs related to AD and available as label 1 are exceedingly partial. To solve this label imbalance problem, we applied a method to randomly select a gene pair corresponding to class label 0 by the size of class label 1.

The number of AD-associated gene pairs established in previous studies was too low to enable effective learning. We used AD-associated gene pairs published by the IntAct database as basic data for class label 1. Additionally, we used an AD-associated gene set curated by many GWAS and included additional gene pairs that can extend the interactome network. The number of AD-associated gene sets was 642. Using those genes as a seed, the corresponding interactions of the first-neighbouring genes from the seed were included in the dataset. In order to determine whether the use of extended interactions from AD-related genes as a set of correct answers is useful, data without the seed were also used to study the model.

**Fig 3:**
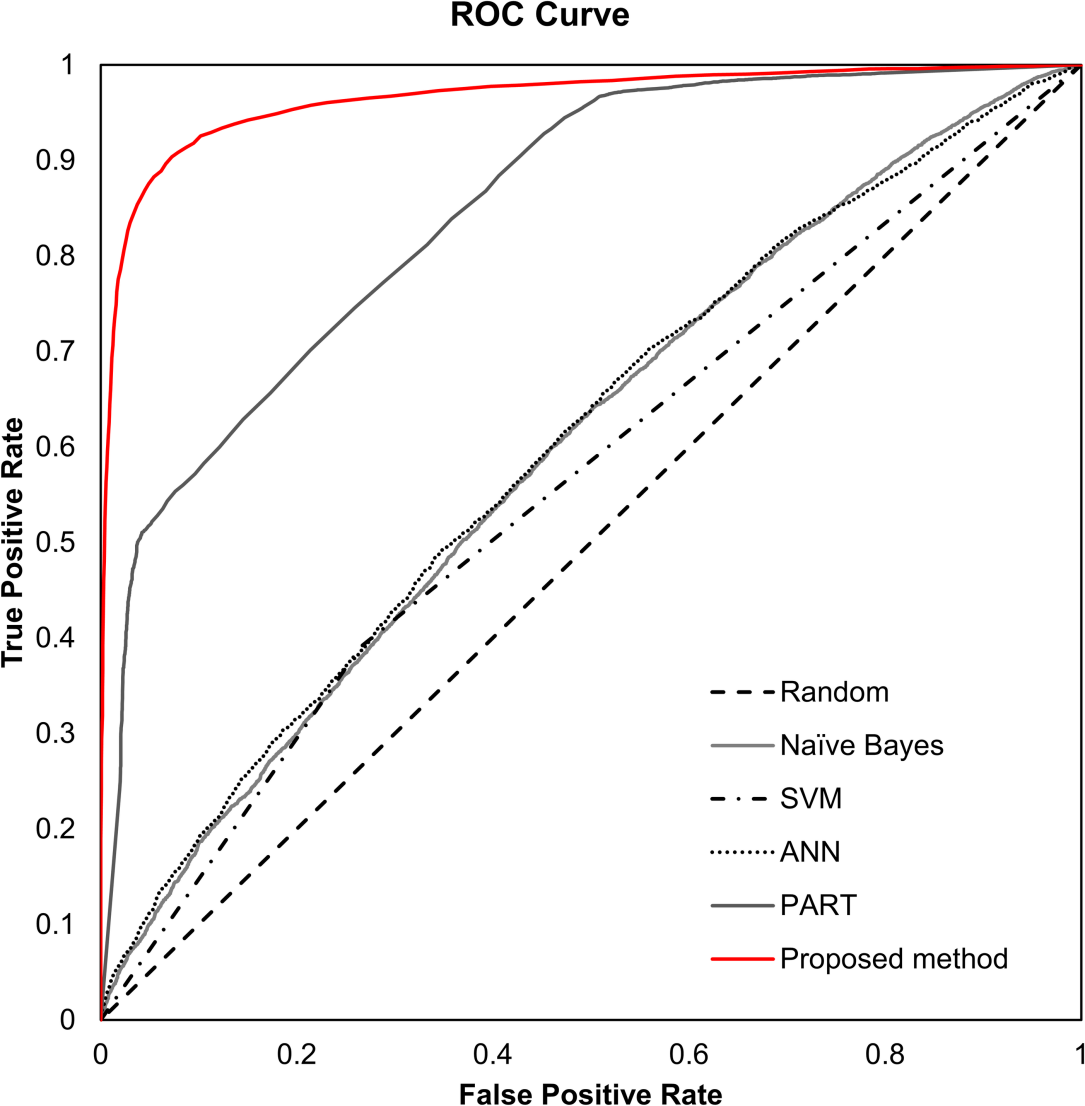
ROC curve for various algorithms using dataset 3.

We compared the proposed approach to four common algorithms using the Weka 3.8 library [31]. The algorithms used for the comparison are listed above with the applied options. 10-fold cross validation was performed to test the performance of algorithms and weighted averages of accuracy, precision, recall, F-measure, and ROC area were obtained. Table 6 summarises the experimental results. For dataset 1, 2, and 3, the proposed method entirely outperformed other algorithms. PART showed the next best performance for these datasets. PART is a rule-based classifier; it combines the divide and conquer strategy with the separate and conquer strategy for rule learning. PART creates a partial decision tree from the training data set to generate the rule. In terms of creating and using a decision tree, PART and the proposed method were similar, but the proposed method uses a bootstrap aggregating approach. We speculated that this approach would improve performance. About three datasets, the accuracy and ROC area values of the proposed method were not significantly different. Nonetheless, when we used dataset 3, the proposed method generally showed the best performance. (Fig 3) shows the ROC curve for the performance comparison using dataset 3. The same comparative analyses were performed for dataset 4, 5, and 6. In these experiments, the proposed method also outperformed the other four algorithms. However, the accuracy and ROC area for the proposed method were relatively lower than those observed for dataset 1, 2, and 3. In order to improve the classification performance of the model, we concluded that it is necessary to use the training data set using the genes known to be related to the disease.

**Table 6:** Comparison of the performance of various algorithms for dataset 1, 2, and 3. The proposed method showed the best performance for all three datasets.

Dataset	Algorithm	Weighted average				
		Accuracy	Precision	Recall	F-Measure	ROC area
1	Naïve Bayes	0.537	0.551	0.537	0.504	0.581
	SVM	0.580	0.580	0.580	0.579	0.580
	ANN	0.570	0.570	0.570	0.570	0.603
	PART	0.742	0.742	0.742	0.742	0.842
	Proposed method	0.902	0.905	0.902	0.902	0.954
2	Naïve Bayes	0.547	0.567	0.547	0.512	0.585
	SVM	0.562	0.564	0.562	0.559	0.562
	ANN	0.567	0.567	0.567	0.567	0.597
	PART	0.713	0.723	0.713	0.710	0.812
	Proposed method	0.898	0.899	0.898	0.898	0.953
3	Naïve Bayes	0.549	0.567	0.549	0.518	0.597
	SVM	0.563	0.571	0.563	0.549	0.563
	ANN	0.570	0.570	0.570	0.570	0.601
	PART	0.744	0.746	0.744	0.743	0.850
	Proposed method	0.916	0.916	0.916	0.916	0.965
4	Naïve Bayes	0.529	0.533	0.529	0.515	0.555
	SVM	0.552	0.552	0.552	0.551	0.552
	ANN	0.535	0.537	0.535	0.528	0.565
	PART	0.628	0.628	0.628	0.628	0.704
	Proposed method	0.783	0.783	0.783	0.782	0.861
5	Naïve Bayes	0.540	0.560	0.540	0.499	0.577
	SVM	0.556	0.580	0.556	0.522	0.556
	ANN	0.559	0.559	0.559	0.559	0.587
	PART	0.642	0.644	0.642	0.640	0.718
	Proposed method	0.772	0.773	0.772	0.772	0.851
6	Naïve Bayes	0.535	0.552	0.535	0.494	0.571
	SVM	0.555	0.583	0.555	0.515	0.555
	ANN	0.565	0.566	0.565	0.565	0.591
	PART	0.662	0.662	0.662	0.662	0.752
	Proposed method	0.786	0.786	0.786	0.786	0.865

### Analysis of features

As shown in (Fig 3), the SVM algorithm, known to have good performance, showed worse performance compared to that of the random forest algorithm. The main difference between these two algorithms is that random forest uses an ensemble learning approach by making multiple decision trees with partial features. SVM uses all 22 features for training. Therefore, we investigated which features were more important in the random forest algorithm, and compared them to the feature lists extracted by typical three-feature selection algorithms. S1 Table shows the comparative results. Interestingly, feature lists obtained from the three algorithms were similar. In particular, as shown in S1 Table, although the order of features was different, the top seven features were the same across the three methods. We also obtained the ranking of features that are important in the random forest algorithm based on average impurity values. We then compared all feature lists among the four cases, as shown in S1 Table. Interestingly, we could find differences in the patterns of the feature rankings. For the random forest, the correlation-based features using PCC and MI were relatively less important than statistic-based features, such as means and standard deviation.

### Application of the proposed approach

To test the applicability of the proposed algorithm, we used another publicly available AD gene expression dataset to classify GGIs. We downloaded a human brain transcript expression dataset from GEO (accession number GSE15222) [32]. This dataset was made to analyse late-onset AD and included 176 normal and 186 AD samples. Of the 176 normal samples, two samples with inaccurate ages were excluded.

Among all possible gene pairs using 360 samples, we focused on the partial gene pairs that exist in the interactome dataset. This ensured that the proposed algorithm extracts biologically meaningful GGIs from gene expression data from completely different platforms. In this experiment, we used bPPI and HumanNet because this was a highly confident dataset for which the physical interactions between the two proteins and the correlations between genes were empirically proven using several techniques. Using this interactome dataset, 22 features were extracted from the expression profile.

**Fig 4:**
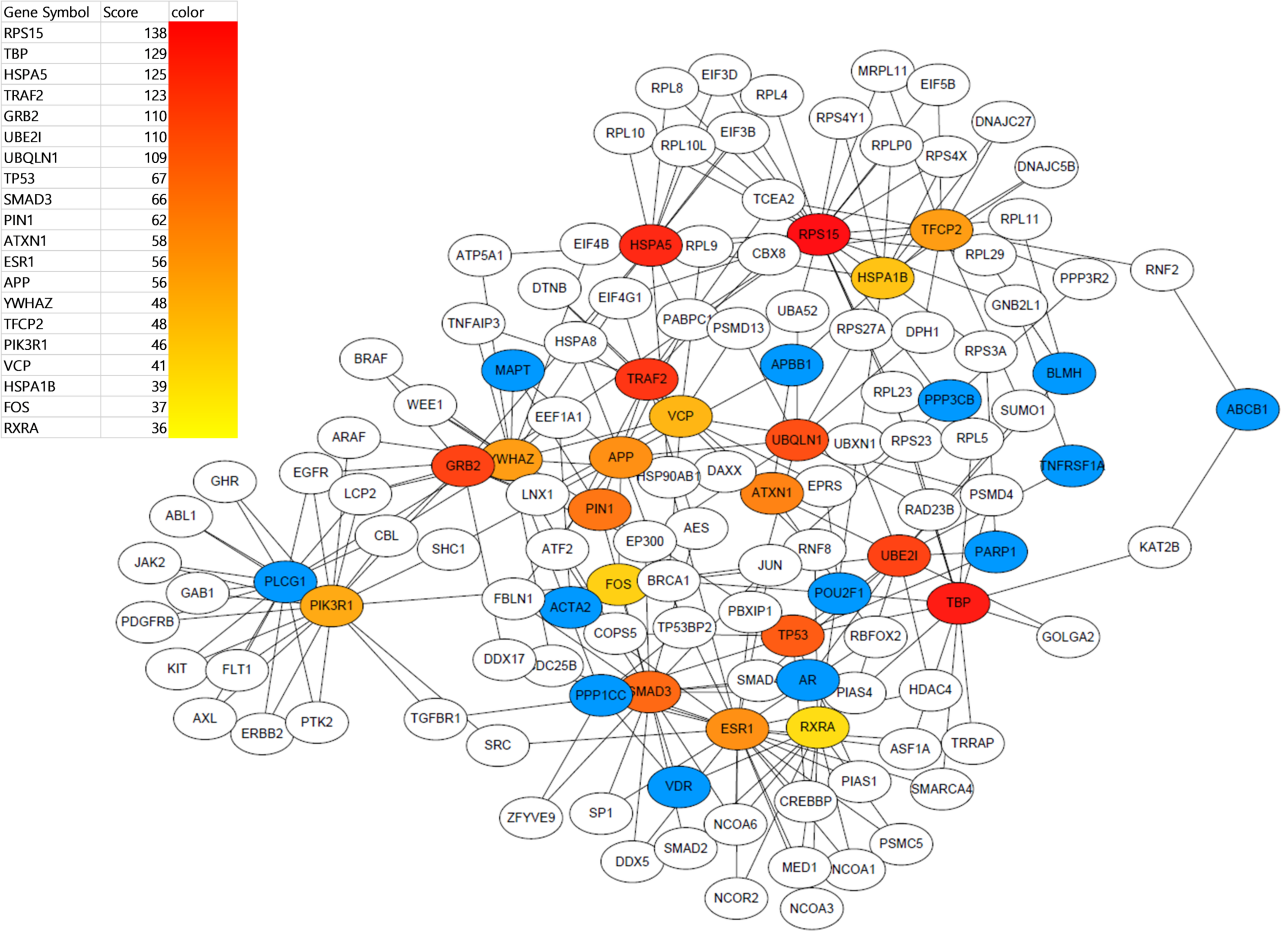
Visualisation of the subnetwork for features extracted by a degree-based topological analysis. The number of nodes and edges were 130 and 247, respectively. The nodes coloured sequentially from red to yellow are the top 20 genes with a high degree. Blue nodes indicate seed genes.

As a result, 3,366 GGIs were identified to be AD-related, i.e. a correlation between two genes was classified by the proposed algorithm after training using dataset 3. We constructed a gene network with the classified GGIs, as shown in S1 Fig. To demonstrate whether the constructed network reflects the AD-related biological context or not, we applied a simple topological analysis. We selected the top 20 genes with high degrees and extracted the subnetwork that can be made from them. To do this, we used Cytoscape with the cytoHubba [33] package. (Fig 4) shows the extracted subnetwork, where reddish nodes represent those with the highest degree. Interestingly, the subnetwork included *APP*, known to be highly related to AD.

**Fig 5:**
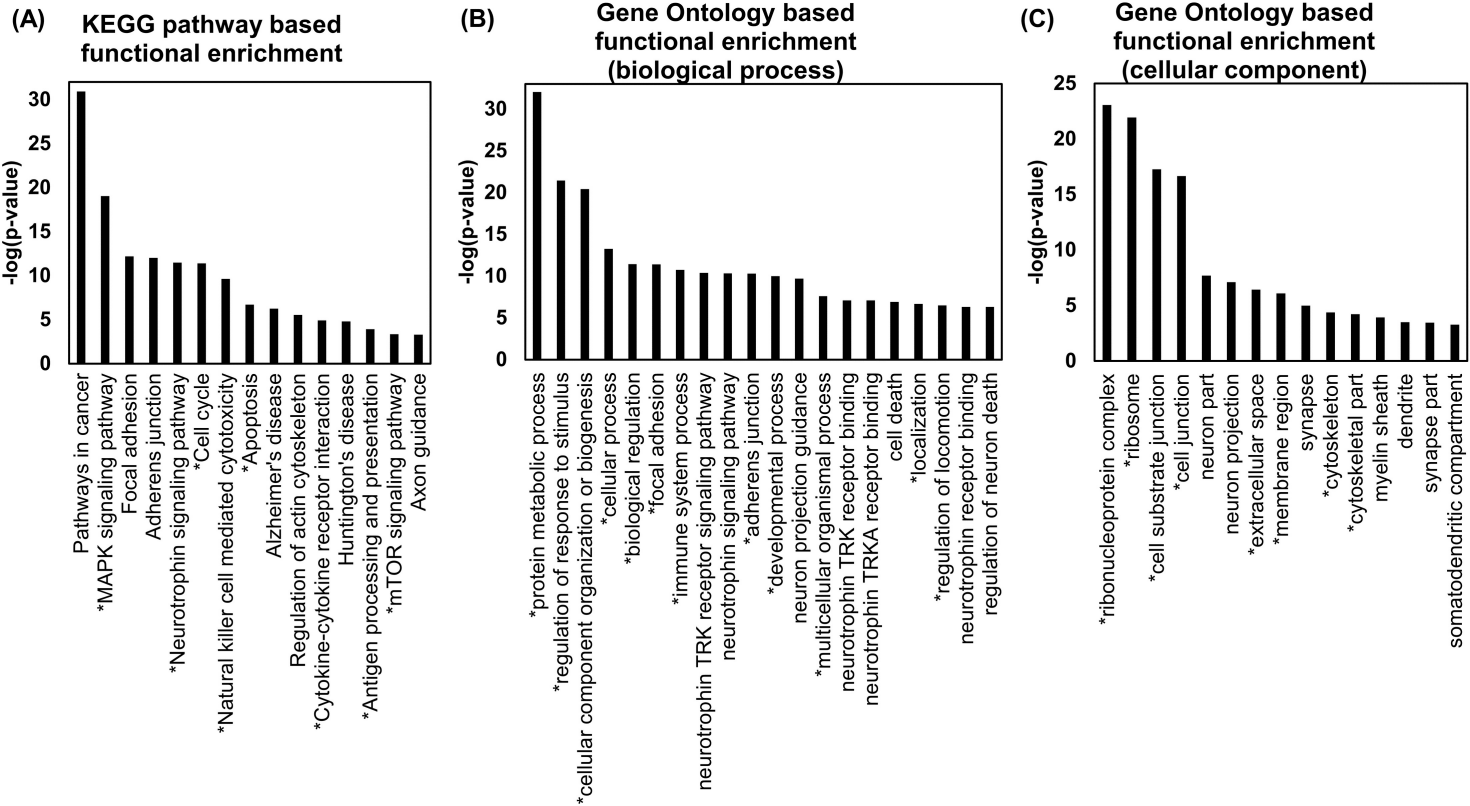
Functional enrichment results for the GSE15222 dataset. An asterisk of a pathway and GO term indicates that it has been reported in previous studies. (A) We used GSEA with a FDR q-value threshold of 0.001 and selected 15 pathways that satisfy the threshold. Interestingly, several AD-related pathways, such as Regulation of actin cytoskeleton and Neurotrophin signalling pathway, were enriched as well as the Alzheimer’s disease pathway. (B) We used FuncAssociates 3.0 with the default evidence code. The p-value threshold was 0.001 and we selected 20 GO terms that are potentially related to AD. We found that many GO terms related to AD were significantly enriched. (C) We used GSEA with a FDR q-value threshold of 0.001 and selected 15 GO terms in the cellular component category that satisfy the threshold and are potentially related to neuronal functions.

We also performed a functional enrichment test for the subnetwork using Gene Set Enrichment Analysis (GSEA) and FuncAssociate 3.0 [34]. The results are shown in (Fig 5). Because the subnetwork contained 130 genes, many pathways and Gene Ontology (GO) terms were enriched, despite applying a strict p-value cutoff of 0.001. Among them, we selected several (15~20) representative results that might be relevant to AD. To investigate whether the enrichment results are relevant to AD, we analysed previous literature.

As shown in (Fig 5(A)), eight pathways marked with an asterisk, such as the MAPK signalling pathway, neurotrophin signalling pathway, cell cycle, Natural killer cell-mediated cytotoxicity, Apoptosis, Cytokine-cytokine receptor interaction, Antigen processing and presentation, and mTOR signalling pathway, have been reported to be related to AD in a previous study [35]. As shown in (Fig 5(B)), 130 genes were significantly related to neuronal cell processes and several basic cellular processes, such as adhesion, developmental processes, and cell death. Twelve GO terms are also related to AD according to a previous study [36]. We identified that many GO terms related to neurons and synapses, including neuron part, synapse, myelin sheath, and dendrite, were significantly enriched, as shown in (Fig 5(C)). These results also confirmed that many GO terms in the cellular component category overlapped with those identified in a previous study [36].

Finally, we investigated correlation values for the GGIs predicted to be AD-related, but lacking from the answer set. Here, the answer set indicates GGIs associated with AD, as shown in Table 4. Let us assume that those GGIs have low correlation coefficients. We tried to demonstrate that such GGIs could not be identified as AD-related by applying typical methods based on correlation measures. Since the proposed method used 22 features derived from the expression profile, it was possible to classify significant GGIs, despite the weak correlations. To verify that sure this assumption was true, we selected GGIs that were classified as AD-related, but did not exist in the answer set. We calculated means and standard deviation of the correlation coefficients, such as the PCC and MI, for these selected GGIs. As expected, as shown in S2 Table, the average PCC and MI values for these GGIs were too low to identify significant associations. We confirmed that the proposed was able to account for heterogeneous gene expression data.

### Discussion

The present study focused on the issue of not extracting correlated GGIs from gene expression profiles owing to heterogeneity in expression levels across samples assigned to the same conditions. This heterogeneity problem has been reported in AD; accordingly, we used an AD-related gene expression dataset. However, since the proposed method is not disease-specific and follows a general data analysis method, it can be applied to cancer and other diseases, in addition to AD.

In addition, the proposed method can be used alone to identify GGIs, but it can also be used with correlation measures, such as PCC or mutual information. For example, if the correlation measure is as high as 0.9, GGIs can be determined without applying the proposed method, and if the GGI cannot be determined based on the correlation measure alone, it can be determined using the classification model. Accordingly, we can collect a large number of expression datasets for each disease, develop a classification model for GGI in advance, and utilise the model.

## Conclusions

We proposed a novel method to identify GGIs from gene expression profiles. We demonstrated that a machine learning approach, especially the random forest algorithm, could be used to discover significant GGIs from heterogeneous gene expression datasets. In this process, we proposed a method to create 22 features from a gene expression profile and to obtain a classification model using an interactome dataset. We evaluated performance with various AD-related datasets and found that the proposed method showed the best performance. In the future, we plan to study whether the proposed method can be applied to additional disease groups to generate truly meaningful gene networks.

## Supporting information

S1 Fig Visualisation of the classified gene network generated using the proposed method for the GSE15222 dataset. The number of nodes and edges were 2,575 and 3,366, respectively. Blue nodes indicate the seed genes, which are known to be related to AD.
(PDF)

S1 Table Comparison of important features among approaches. In the priority list of features selected through the three algorithms, the seven highest ranked features were the same, but differed with respect to order. These top seven features are indicated with 4 different colours. For the top seven features, we confirmed that the results of the three feature selection algorithms are the same except for the priority. However, the priority of features changed overall in Random Forest.
(DOCX)

S2 Table Basic statistical summary of correlations for gene pairs that are predicted, but absent from the answer set. (DOCX)

S1 File Supporting method is included in this file. (DOCX)
