# Supplementary figures and images for: Machine learning-based identification of genetic interactions from heterogeneous gene expression profiles
Source: PLoS One. 2018 Jul 26;13(7):e0201056. doi: 10.1371/journal.pone.0201056 (PMC6062065; doi:10.1371/journal.pone.0201056)

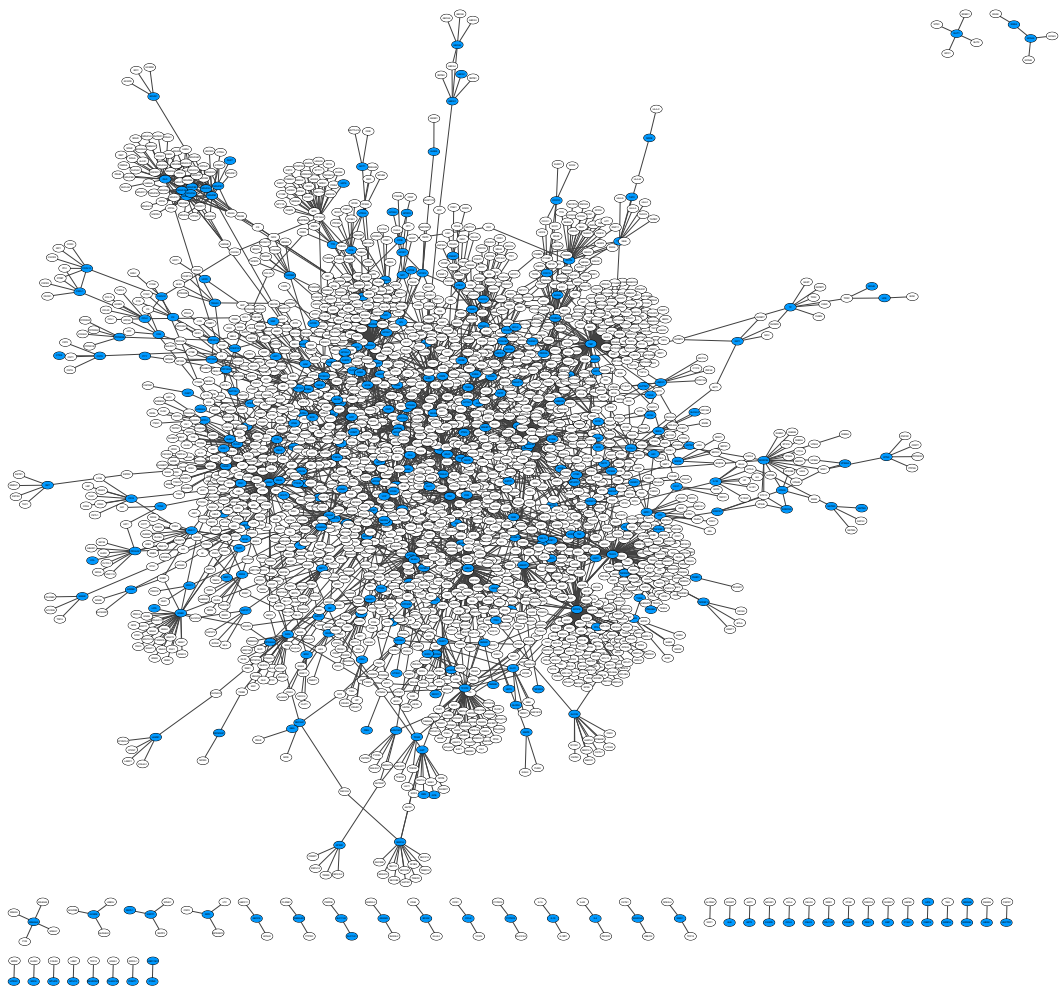

Supplement: S1 Fig — The number of nodes and edges were 2,575 and 3,366, respectively. Blue nodes indicate the seed genes, which are known to be related to AD. (PDF) [file pone.0201056.s001.pdf]
