# Supplementary material for: Machine learning-based identification of genetic interactions from heterogeneous gene expression profiles
Source: PLoS One. 2018 Jul 26;13(7):e0201056. doi: 10.1371/journal.pone.0201056 (PMC6062065; doi:10.1371/journal.pone.0201056)
Supplement: S1 Table — In the priority list of features selected through the three algorithms, the seven highest ranked features were the same, but differed with respect to order. These top seven features are indicated with 4 different colours. For the top seven features, we confirmed that the results of the three feature selection algorithms are the same except for the priority. However, the priority of features changed overall in Random Forest. (DOCX) [file pone.0201056.s002.docx]

S1 Table. Comparison of important features among approaches. In the priority list of features selected through the three algorithms, the seven highest ranked features were the same, but differed with respect to order. These top seven features are indicated with 4 different colours. For the top seven features, we confirmed that the results of the three feature selection algorithms are the same except for the priority. However, the priority of features changed overall in Random Forest.

| Priority | Feature selection method | | | Random Forest  (important attribute based on average impurity decrease) |
| --- | --- | --- | --- | --- |
|  | RELIEFF | Information Gain | Gain Ratio |  |
| 1 | WT_A_L0_A_L1_ | Mean_A_L0_ | SD_A_L0_ | Mean_B_L0_ |
| 2 | SD_A_L0_ | dMm_A_L1_ | Mean_A_L0_ | Mean_B_L1_ |
| 3 | dMm_A_L1_ | dMm_A_L0_ | Mean_A_L1_ | Mean_A_L0_ |
| 4 | SD_A_L1_ | Mean_A_L1_ | dMm_A_L0_ | SD_B_L0_ |
| 5 | dMm_A_L0_ | SD_A_L0_ | dMm_A_L1_ | SD_B_L1_ |
| 6 | Mean_A_L1_ | WT_A_L0_A_L1_ | WT_A_L0_A_L1_ | Mean_A_L1_ |
| 7 | Mean_A_L0_ | SD_A_L1_ | SD_A_L1_ | dMm_B_L0_ |
| 8 | WT_A_L0_B_L0_ | MI_A_L0_B_L0_ | dMm_B_L1_ | SD_A_L0_ |
| 9 | MI_A_L0_B_L0_ | MI_A_L1_B_L1_ | dMm_B_L0_ | dMm_B_L1_ |
| 10 | WT_A_L1_B_L1_ | PCC_A_L0_B_L0_ | PCC_A_L1_B_L1_ | SD_A_L1_ |
| 11 | MI_A_L1_B_L1_ | PCC_A_L1_B_L1_ | PCC_A_L0_B_L0_ | WT_A_L0_B_L0_ |
| 12 | PCC_A_L0_B_L0_ | WT_B_L0_B_L1_ | Mean_B_L0_ | WT_A_L1_B_L1_ |
| 13 | MI_A_L0_A_L1_ | MI_A_L0_A_L1_ | MI_A_L1_B_L1_ | PCC_A_L0_B_L0_ |
| 14 | dMm_B_L1_ | Mean_B_L0_ | MI_A_L0_B_L0_ | WT_B_L0_B_L1_ |
| 15 | PCC_A_L1_B_L1_ | Mean_B_L1_ | MI_A_L0_A_L1_ | dMm_A_L0_ |
| 16 | Mean_B_L1_ | SD_B_L0_ | WT_B_L0_B_L1_ | MI_B_L0_B_L1_ |
| 17 | Mean_B_L0_ | dMm_B_L0_ | SD_B_L0_ | dMm_A_L1_ |
| 18 | SD_B_L1_ | MI_B_L0_B_L1_ | Mean_B_L1_ | PCC_A_L1_B_L1_ |
| 19 | dMm_B_L0_ | SD_B_L1_ | MI_B_L0_B_L1_ | MI_A_L1_B_L1_ |
| 20 | SD_B_L0_ | dMm_B_L1_ | SD_B_L1_ | MI_A_L0_B_L0_ |
| 21 | WT_B_L0_B_L1_ | WT_A_L1_B_L1_ | WT_A_L0_B_L0_ | MI_A_L0_A_L1_ |
| 22 | MI_B_L0_B_L1_ | WT_A_L0_B_L0_ | WT_A_L1_B_L1_ | WT_A_L0_A_L1_ |
