# Supplementary material for: Machine learning-based identification of genetic interactions from heterogeneous gene expression profiles
Source: PLoS One. 2018 Jul 26;13(7):e0201056. doi: 10.1371/journal.pone.0201056 (PMC6062065; doi:10.1371/journal.pone.0201056)
Supplement: S2 Table — (DOCX) [file pone.0201056.s003.docx]

S2 Table. Basic statistical summary of correlations for gene pairs that are predicted, but absent from the answer set.

| Class | Correlation measure | Mean | SD |
| --- | --- | --- | --- |
| Normal | PCC | -0.075 | 0.377 |
|  | MI | 0.178 | 0.126 |
| AD | PCC | -0.064 | 0.373 |
|  | MI | 0.192 | 0.125 |
