# Supplementary material for: Machine learning-based identification of genetic interactions from heterogeneous gene expression profiles
Source: PLoS One. 2018 Jul 26;13(7):e0201056. doi: 10.1371/journal.pone.0201056 (PMC6062065; doi:10.1371/journal.pone.0201056)
Supplement: S1 File — (DOCX) [file pone.0201056.s004.docx]

**Machine Learning-based Identification of Genetic Interactions from Heterogeneous Gene Expression Profiles**

Chihyun Park^1^, JungRim Kim^1^, Jeongwoo Kim^1^, and Sanghyun Park^1^*

^1^ Dept. of Computer Science, Yonsei University, 134, Shinchon-dong, Seodaemun-gu, Seoul, Korea

*Corresponding author

E-mail: [sanghyun@yonsei.ac.kr](mailto:sanghyun@yonsei.ac.kr)

**Supporting method**

**Calculating Welch’s *t*-test statistics and Mutual Information**

As described in Methods section of the manuscript, gene expression profile of each gene pair can be represented as follows.

| Gene pairs | Class label 0 (Normal) | Class label 1 (AD) |
| --- | --- | --- |
| Gene A | E_A_L0_ | E_A_L1_ |
| Gene B | E_B_L0_ | E_B_L1_ |

With these four expression value lists, we calculated Welch’s *t*-test statistics according to the following formula.

$$\mathrm{WT}\left( X_{1}, X_{2} \right)= \frac{\bar{X_{1}}-\bar{X_{2}}}{\sqrt{\frac{s_{1}^{2}}{N_{1}}+\frac{s_{2}^{2}}{N_{2}}}}$$

*X*_1_ and *X*_2_ indicate the input value lists. And $\bar{X}$ and *S*^2^ are the mean and variance of *X*. *N* is the size of the value list *X*. For four pairs of element lists, we calculate the statistics. Because the statistics can be computed even if the lengths of the lists are different, we calculate the value for two lists with different class labels.

We calculate Mutual Information through the method proposed in the previous paper [1]. Mutual Information requires binning for the continuous dataset. The previous study have suggested a method to calculate Mutual Information based on b-spline method from gene expression data. It was showed this approach was more effective when data is noisy, such as gene expression measurement. We adopted the method to calculate Mutual Information.

**References**

1. Park C, Yoon Y, Oh M, Yu SJ, Ahn J. Systematic identification of differential gene network to elucidate Alzheimer's disease. Expert Syst. Appl. 2017;85: 249-260.
